# Metabolic syndrome and the immunogenicity of Pfizer–BioNTech vaccine: a cross-sectional study in Japanese healthcare workers

**DOI:** 10.1186/s13098-022-00918-6

**Published:** 2022-10-13

**Authors:** Dong Van Hoang, Shohei Yamamoto, Ami Fukunaga, Yosuke Inoue, Tetsuya Mizoue, Norio Ohmagari

**Affiliations:** 1 Department of Epidemiology and Prevention, Center for Clinical Sciences, National Center for Global Health and Medicine, Toyama 1-21-1, Shinjuku, Tokyo, Japan; 2 Disease Control and Prevention Center, National Center for Global Health and Medicine, Toyama 1-21-1, Shinjuku, Tokyo, Japan

**Keywords:** Metabolic syndrome; immunogenicity, Pfizer-BioNTech, Vaccine, Japan

## Abstract

**Background:**

The clustering of metabolic abnormalities may weaken vaccine-induced immunity, but epidemiological data regarding SARS-CoV-2 vaccines are scarce. The present study aimed to examine the cross-sectional association between metabolic syndrome (MetS) and humoral immune response to Pfizer–BioNTech vaccine among the staff of a research center for medical care in Japan.

**Methods:**

Participants were the staff (aged 21–75 years) of the National Center of Global Health and Medicine who had completed the second dose of Pfizer–BioNTech vaccine 1–3 months before the survey. MetS was defined according to the Joint Interim Statement. SARS-CoV-2 spike immunoglobulin G (IgG) antibody was measured using quantitative assays. Multivariable linear regression was used to estimate the geometric mean titers (GMT) and geometric mean ratio (GMR) of IgG titers, relative to MetS status.

**Results:**

Of 946 participants who received the second vaccine dose, 51 (5.4%) had MetS. Those with MetS had a significantly lower IgG titer (GMT 4125; 95% confidence interval [CI], 2885–5896) than those without MetS (GMT 5348; 95% CI, 3914–7309); the GMR was 0.77 (95% CI 0.64–0.93). Taking those having no MetS component as reference, fully adjusted GMR (95% CI) for those having 1, 2, 3 or ≥ 4 components was 1.00 (0.90, 1.11), 0.89 (0.77, 1.04), 0.86 (0.68, 1.10) and 0.61 (0.45, 0.82), respectively (P trend = 0.024).

**Conclusion:**

Results suggest that having MetS and a greater number of its components are associated with a weaker humoral immune response to the Pfizer–BioNTech vaccine.

**Supplementary Information:**

The online version contains supplementary material available at 10.1186/s13098-022-00918-6.

## Background

The ongoing pandemic of coronavirus disease 2019 (COVID-19), caused by severe acute respiratory syndrome coronavirus (SARS-CoV-2), has posed a serious threat to global health, with more than 450 million patients and more than 6 million deaths [1]. Immunization with SARS-CoV-2 vaccines is a global strategy to minimize deaths, severity, and overall disease burden of the pandemic [2]. While most recommended SARS-CoV-2 vaccines, such as BioNTech (BNT162b2) and Moderna (mRNA-1273) can achieve high efficacy [3], their immunogenicity can be hampered by several factors, e.g., aging, virus mutation [4, 5], smoking [5], obesity [6, 7], diabetes [7], and other underlying comorbidities [5]. Identification of such factors may be of public health significance regarding the prevention of the virus infection, e.g., administration of an earlier vaccine booster in high-risk groups [8].

Epidemiological data suggest that metabolic syndrome (MetS), a major public health concern for many countries worldwide [9, 10], may hamper the humoral response to SARS-CoV-2 vaccines. More specifically, MetS can lead to a chronic inflammatory state (e.g., increased circulating adipokines and cytokine-like hormones) which in turn may result in a decrease in immunogenicity following vaccination [11–13]. A few studies[7, 14, 15] showed that individual MetS components may reduce the immune response to SARS-CoV-2 vaccines. For example, central obesity was associated with lower immunoglobulin (Ig) G antibody titers [14]; while diabetes was inversely associated with IgG antibody concentration [15], after the vaccination with Pfizer–BioNTech. However, we are not aware of any epidemiological data linking MetS to immune response to SARS-CoV-2 vaccines. We hypothesize that MetS is associated with a weaker humoral response to the SARS-CoV-2 vaccine. The present study aimed to examine the association between MetS and SARS-CoV-2 spike IgG antibody titer among recipients of two doses of the Pfizer–BioNTech vaccine.

## Method

### Study setting and participants

Since July 2020, a repeatitive serological survey has been being conducted to monitor the spread of SARS-CoV-2 infection among the staff members of the National Center for Global Health and Medicine (NCGM), Japan [16–18]. Participants were asked to donate a blood sample for the measurement of anti-SARS-CoV-2 antibodies. We also collected the information on medical history, health-related lifestyle, and COVID-19 (e.g., COVID-19 infection and vaccination) via an online questionnaire. The participation in the survey was completely voluntary; and a written informed consent was obtained from each participant.

In the present study, we used data of the third round of survey conducted in June 2021, 2 months after the completion of an in-house vaccination program (Pfizer–BioNTech). We additionally obtained annual health check-up information which was collected in the same year as the survey (June 2021). Eligible participants were NCGM’s staff of all occupations (including doctors, nurses, administrative staff, and allied healthcare professionals) who had completed two doses of the vaccine. We excluded those who disagreed to provide their health check-up data, received antibody test within 14 days of the second vaccination, or lacked information on MetS components or covariates.

### Assessment of SARS-CoV-2 antibodies

We quantitatively measured IgG (AU/mL) against the SARS-CoV-2 spike protein, using AdviseDx SARS-CoV-2 IgG II assay, Abbott ARCHITECT^®^. In a subgroup of the vaccine recipients (n = 68), the Spearman’s rank correlation coefficient (95% CI) between the above SARS-CoV-2 spike IgG titer and neutralizing antibody titer was 0.497 (0.286–0.661), 0.250 (0.005–0.467), and 0.683 (0.526–0.795) against Wuhan, Alpha, and Delta strains, respectively. We also qualitatively measured antibodies against SARS-CoV-2 nucleocapsid protein using the SARS-CoV-2 IgG assay (Abbott) and used these data to identify those with possible infection. The sensitivity and specificity for the identification of past infection with SARS-CoV-2 viruses using this assay were 100% and 99.9%, respectively [19].

### Assessment of metabolic syndrome and covariates

The information on MetS components, i.e., waist circumference (WC), blood pressure (BP), fasting plasma glucose (FPG), triglycerides (TG), and high-density lipoprotein cholesterol (HDL-C), was collected during the health check-up. WC was measured at the umbilical level in a standing position using a measuring tape (maximum:150 cm); Systolic and diastolic BP were measured with an automated sphygmomanometer (HEM-907, Omron Health Care Co. Ltd., Kyoto, Japan); FPG was measured using an enzymatic (Hexokinase UV) method (Cica Liquid GLU, Kanto Chemical Co., Tokyo, Japan); TG level was measured by an enzymatic method using the Pureauto S TG-N (Sekisui Medical Co., Ltd., Tokyo, Japan); and HDL-C concentration was measured by a direct enzymatic method using the Cholestest-N HDL (Sekisui Medical Co., Ltd., Tokyo, Japan).

MetS was defined, according to the Joint Interim Statement [20], as a clustering of any three or more of the following components: high FPG (≥ 100 mg/dL or using anti-diabetic medication), central obesity (WC ≥ 90 cm for men, or ≥ 80 cm for women), high TG (≥ 150 mg/dL or using lipid-lowering medication), high BP (systolic BP ≥ 130 mmHg, diastolic BP ≥ 85 mmHg or using antihypertensive medication) and reduced HDL-C (< 40 mg/dL for men or < 50 mg/dL for women). The cut-off values for WC were based on the recommendation of the World Health Organization for Asian populations [21].

We selected covariates according to epidemiological evidence for their association with the immune response to SARS-CoV-2 vaccines: age, sex [4, 5], smoking [5], alcohol drinking [22], physical activity [23], underlying comorbidities (i.e., cancer, heart, or lung diseases) [5, 24], history of SARS-CoV-2 infection [22, 25], and the time interval (in day) between the second dose of SARS-CoV-2 vaccine and the day of blood draw (vaccination-to-IgG time) [22]. The history of infection with SARS-CoV-2 was defined as the positive result of either polymerase chain reaction test or antibodies against SARS-CoV-2 nucleocapsid protein.

### Statistical analysis

The background characteristics of the study population, according to MetS status, were described as arithmetic mean and standard deviation (SD), or median and range/interquantile range for continuous variables, and percentages for categorical variables.

Linear regression modeling was used to estimate the means (95% confidence interval [CI]), and the beta-coefficients (95% CI) of log_10_-transformed SARS-CoV-2 spike IgG titers, relative to MetS. Two models were fitted: Model 1 was adjusted for age and sex; and Model 2 was further adjusted for smoking (non-smoker, or smoker), alcohol drinking (non-drinker, drinker consuming < 23 or ≥ 23 g ethanol/day), leisure-time physical activity (non-engagement, < 150, or ≥ 150 min/week), comorbid cancer (all types), heart or lung diseases, history of SARS-CoV-2 infection, and vaccination-to-IgG time. The marginal means (95% CI) predicted from Model 2 were then back-transformed to obtain the adjusted geometric mean titer (GMT) (95% CI) of SARS-CoV-2 spike IgG. The beta-coefficients (95% CI) from Model 2 were back-transformed to obtain the geometric mean ratio (GMR) (95% CI) for SARS-CoV-2 spike IgG titer.

We also examined the association between the number of MetS components and SARS-CoV-2 spike IgG titers, using Model 1 and Model 2 in which those with five components were regrouped together with those having four components. The trend in this association was assessed by assigning an ordinal number (1–5) to each group, which was treated as a continuous variable when fitted in regression models.

To eliminate the potential impact of comorbidities and history of SARS-CoV-2 infection on the association between MetS and the immunogenicity of Pfizer–BioNTech vaccine, we conducted a sensitivity analysis using Model 1 and Model 2 in which we excluded participants with either condition. Statistical significance was set at p < 0.05 for trend and p < 0.1 for interaction tests. All statistical analyses were conducted in RStudio (version 3.2.4) using the package “emmeans” (version 1.6.3) [26].

## Results

**Fig. 1:**
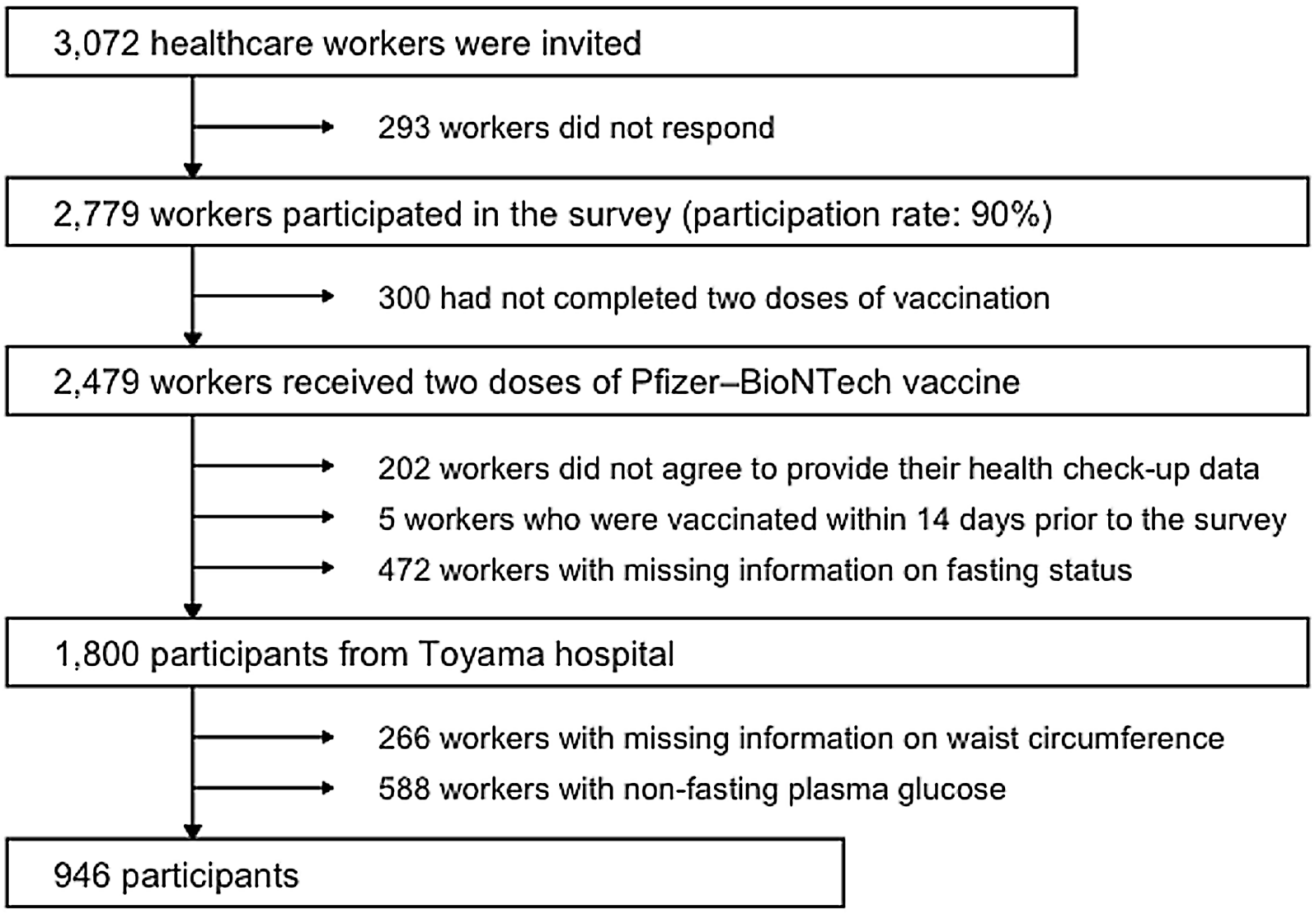
Participant selection

Of 3,072 workers invited, 2779 (90%) agreed to participate. Of these, 2479 had received two doses of Pfizer–BioNTech vaccine. We excluded those who disagreed to provide their health check-up data (n = 202), who received the second vaccination within 14 days prior to the survey (n = 5), who had missing information on fasting status before testing for plasma glucose (n = 472) or WC (n = 266), or those with non-FPG (n = 588), leaving 946 for analysis (Fig. 1). The excluded participants were older and more likely to be women and smokers, and had higher prevalence of comorbidities and history of SARS-Cov-2 infection (Additional file 1: Table S1).

A total of 51 participants (5.4%) had MesS. As shown in Table 1, participants with MetS were older, and more likely to be men, current smokers, and alcohol drinkers, as compared with those without MetS. They had higher prevalence of comorbidities.

**Table 1:** Characteristics of study participants

Characteristics	All participants	Metabolic syndrome		
		No	Yes	P values
N	946	895	51	
Age (year), mean (SD)	36.7 (12.3)	35.9 (12.0)	49.5 (10.7)	< 0.001
Sex (men)	298 (31.5)	271 (30.3)	27 (52.9)	0.001
Smoking				
Non-smoker	855 (90.4)	819 (91.5)	36 (70.6)	< 0.001
Smoker	91 (9.6)	76 (8.5)	15 (29.4)	
Occupation				
Nurse	285 (30.1)	279 (31.2)	6 (11.8)	0.033
Doctor	183 (19.3)	171 (19.0)	12 (23.5)	
Administrative staff	134 (14.2)	125 (14.0)	9 (17.6)	
Allied healthcare professionals	125 (13.2)	119 (13.3)	6 (11.8)	
Others	219 (23.2)	201 (22.5)	18 (35.3)	
Alcohol consumption				
Non-drinker	355 (37.5)	332 (37.1)	23 (45.1)	0.24
Drinker consuming				
< 23 g ethanol/day	442 (46.7)	424 (47.4)	18 (35.3)	
≥ 23 g ethanol/day	149 (15.8)	139 (15.5)	10 (19.6)	
Leisure time physical activity				
Non-engagement	197 (20.8)	185 (20.7)	12 (23.5)	0.49
< 150 min/week	657 (69.5)	625 (69.8)	32 (62.7)	
≥ 150 min/week	92 (9.7)	85 (9.5)	7 (13.7)	
Comorbidities (any of the below)	29 (3.1)	25 (2.8)	4 (7.8)	0.11
Lung disease	18 (1.9)	16 (1.8)	2 (3.9)	0.57
Heart disease	5 (0.5)	4 (0.4)	1 (2.0)	0.64
Cancer	6 (0.6)	5 (0.6)	1 (2.0)	0.75
History of SARS-Cov-2 infection ^a^	5 (0.5)	5 (0.6)	0 (0.0)	-
Vaccine-to-IgG time, median (range) ^b^	67 (15–103)	67 (15–103)	69 (35–98)	0.009
SARS-Cov-2 spike antibody titer (AU/mL), median (P25-P75)	5588 (3346, 9550)	5746 (3443, 9736)	2986 (1729, 5973)	< 0.001
Body mass index (kg/m^2^), mean (SD)	21.6 (3.2)	21.4 (3.0)	26.6 (3.7)	< 0.001
Waist circumference (cm), mean (SD)	76.5 (10.0)	75.6 (9.3)	92.8 (9.4)	< 0.001
Systolic blood pressure (mm Hg), mean (SD)	117.4 (13.0)	116.7 (12.7)	130.4 (11.6)	< 0.001
Diastolic blood pressure (mm Hg), mean (SD)	69.4 (10.0)	68.9 (9.7)	79.2 (9.9)	< 0.001
Fasting blood glucose (mg/dL), mean (SD)	86.6 (16.2)	85.0 (7.7)	114.5 (55.2)	< 0.001
High-density lipoprotein cholesterol (mg/dL), mean (SD)	70.1 (15.4)	70.9 (15.0)	55.9 (15.7)	< 0.001
Triglycerides (mg/dL), median (P25-P75)	62.5 (44.0, 88.8)	61.0 (44.0, 85.0)	167.0 (100.5, 221.0)	< 0.001
Use of lipid-lowering medication, n (%)	38 (4.0)	14 (1.6)	24 (47.1)	< 0.001
Use of antihypertensive medication, n (%)	50 (5.3)	24 (2.7)	26 (51.0)	< 0.001
Use of antidiabetic medication, n (%)	14 (1.5)	2 (0.2)	12 (23.5)	< 0.001

Values are n (%), unless otherwise stated

*P25-P75* 25th–75th percentile range, *AU* antibody unit, *SD* standard deviation

^a^defined as the positive result of either polymerase chain reaction test or the measurement of antibodies against SARS-CoV-2 nucleocapsid protein

^b^time interval (in day) between the second dose of vaccine and the day of blood draw; P values obtained from t-tesst/ Chi-squared test

**Fig. 2:**
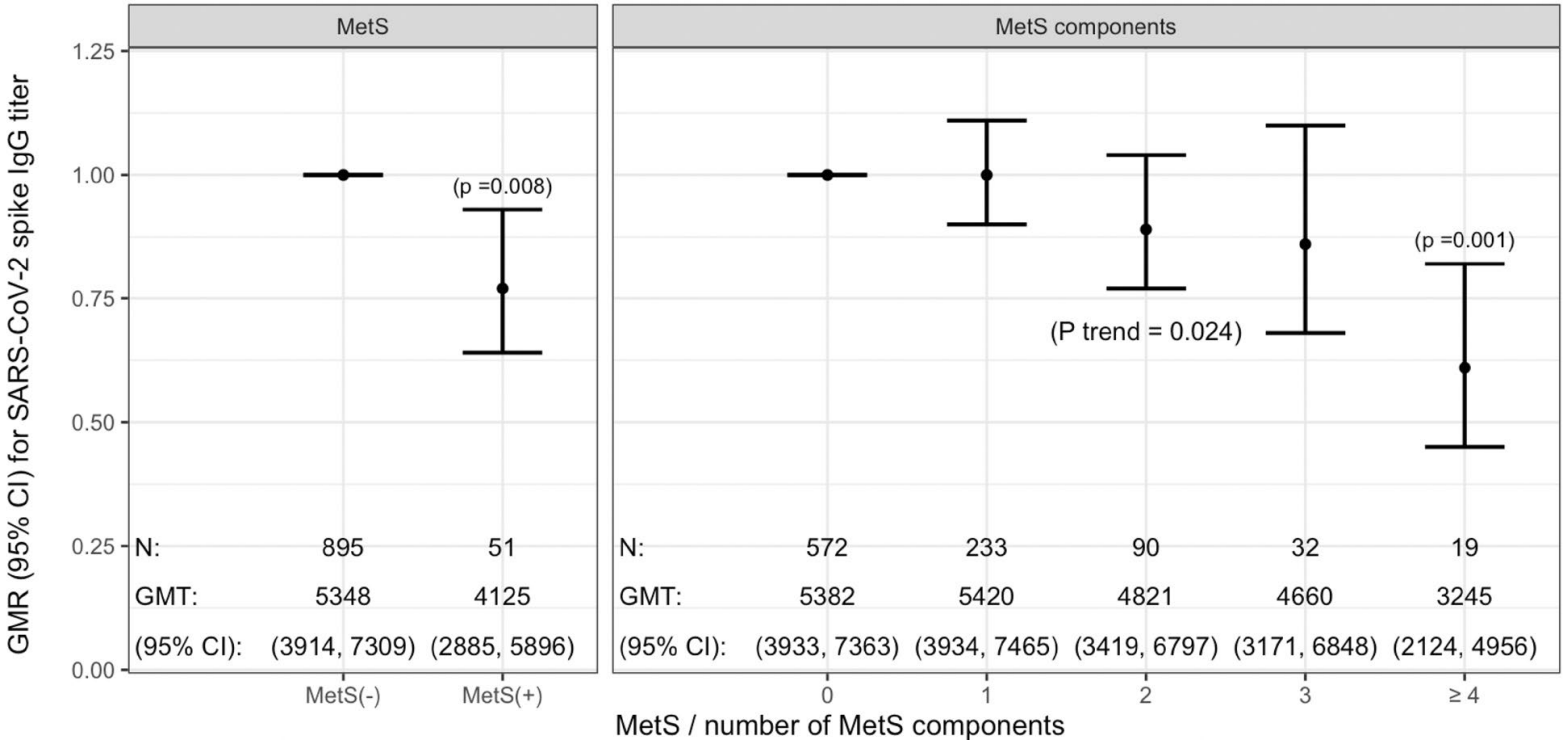
Association between MetS and SARS-CoV-2 spike IgG titer. *MetS* metabolic syndrome; *GMT* geometric mean titer; *GMR* geometric mean ratio; *CI* confidence interval

As shown in Fig. 2, MetS was associated with significantly lower SARS-CoV-2 spike IgG titer. Age- and sex-adjusted GMR (95% CI) was 0.77 (0.63–0.94) for those with MetS. the association remained virtually unchanged (GMR 0.77; 95% CI, 0.64–0.93) after further adjustment for smoking, alcohol consumption, physical activity, history of SARS-CoV-2 infection, duration of time between vaccination and antibody testing, and comorbidity. There was also a significant inverse association between the number of MetS components and SARS-CoV-2 spike IgG titer. Taking those having no MetS component as reference, fully adjusted GMR (95% CI) for those having 1, 2, 3 or ≥ 4 components was 1.00 (0.90, 1.11), 0.89 (0.77, 1.04), 0.86 (0.68, 1.10) and 0.61 (0.45, 0.82), respectively (P_trend_ = 0.024). Further details on beta-coefficients and model fit are presented in Additional file 1: Table S2. In the sensitivity analysis excluding those with a history of SARS-CoV-2 infection or those with comorbid cancer, heart diseases, or lung diseases (n = 34), the results were virtually unchanged; the fully adjusted GMR (95% CI) for MetS was 0.76 (0.62, 0.92) (Additional file 1: Table S3).

## Discussion

In the present cross-sectional study, MetS was associated with a significantly lower SARS-CoV-2 spike IgG antibody titer among healthcare workers who received two doses of Pfizer–BioNTech vaccine. There was also a significant inverse association between the number of MetS components and SARS-CoV-2 spike IgG antibody titer.

We are not aware of any previous studies on the relationship between MetS and SARS-CoV-2 spike IgG antibody titers. Nevertheless, our findings are in line with epidemiological data on the association between individual MetS components and the humoral immunogenicity of SARS-CoV-2 vaccines. For example, in Italian healthcare workers, central obesity (p = 0.026) [14], overweight (p = 0.04) [27], and dyslipidemia (p = 0.005) were each associated with lower IgG antibody titers following Pfizer–BioNTech vaccination. A systematic review of eight studies showed that diabetic patients had weaker immunogenicity following SARS-CoV-2 vaccines (i.e., Pfizer–BioNTech, CoronaVac, and Covishield™), compared with healthy people [15]. In the present study, we observed an inverse dose–response association between the number of MetS components and Pfizer–BioNTech-induced antibody titers, which further consolidated the association between MetS and the weaker immunogenicity of this vaccine.

The mechanism linking MetS to weaker immune response to vaccine is not clear. In the condition of MetS, several pro-inflammatory cytokines (e.g., leptin, TNF-α, and IL-6) are over-secreted, while some anti-inflammatory cytokines (e.g., adiponectin) are under-secreted [12]. This dysregulated production of adipokines results in chronic low-grade inflammation, which in turn may lead to an alteration in the function of B cells, and a subsequent reduction in vaccine-induced antibody production [11, 12, 28, 29]. Impaired glucose metabolism may also contribute to the reduced immune response. For example, hyperglycemia can promote the senescence of CD4 + and CD8 + T cells [30], resulting in reduced immunity of the B cells. Insulin resistance may also hamper the vaccine-induced immunity since it contribute to deteriorate the regulation of T cells’ proliferation and immunity [31].

This study has some limitations. First, we excluded a large number of participants who lacked data on fasting status, FPG, or WC. Nonetheless, these exclusions might not have caused serious bias because they were largely related to logistic reason (e.g., information on fasting status was not collected in one study site). Second, there was a large difference in the distribution of vaccination-to-IgG time between those with MetS and those without Mets. We confirmed, however, that mean SARS-CoV-2 spike IgG titer was consistently lower in those with MetS than in those without MetS across vaccination-to-IgG time (Additional file 1: Figure S1). Third, we did not have information on immunosuppressive medication, which might have confounded the association. Nevertheless, the study participants were healthy workers and few were assumed to have a disease requiring such medication (for instance, only 6 participants had cancer). We also confirmed that the results were materially unchanged after excluding those with cancer, heart or lung diseases, or history of SARS-CoV-2 infection. Fourth, we did not examine the cell-mediated immunogenicity. A study found no association of hypertension and dyslipidemia, components of MetS, with post-vaccine cell-mediated immunogenicity [32]. The effect of MetS on vaccine-induced cellular response may thus differ from what we observed for humoral response. Finally, the present study was done among health care staff with a low prevalence of MetS (5.4%), which is considerably lower than those reported in other industries: mining (20.5%), construction (21.0%), and transportation (25.7%) [32]. Therefore, the generalization of the findings should be made with caution.

## Conclusions

In this cross-sectional study among the staff of a research center for medical care in Japan, MetS was associated with significantly lower concentrations of post-vaccine SARS-CoV-2 spike IgG antibody titers. While logitudinal studies with a larger sample size are required to confirm the observed association, the present results may inform policy makers in formulating the preventive strategy against COVID-19, including the recommendation of earlier booster vaccination for those with MetS.

## Supplementary Information

**Additional file 1:** **Table S1.** Characteristics of included versus excluded participants. **Table S2.** Association between MetS and SARS-Cov-2 spike IgG titers. **Table S3.** Association between MetS and SARS-Cov-2 IgG titers after excluding those with SARS-Cov-2 infection, or comorbid cancer, heart or lung diseases (N = 912).** Figure S1.** Distribution of SARS-CoV-2 spike IgG titer across vaccination-to-IgG time

## Abbreviations

COVID-19: Coronavirus disease 2019
MetS: Metabolic syndrome
Ig: Immunoglobulin
GMT: Geometric mean titer
GMR: Geometric mean ratio
CI: Confidence interval
SARS-CoV-2: Severe acute respiratory syndrome coronavirus
FPG: Fasting plasma glucose
BP: Blood pressure
TG: Triglycerides
HDL-C: High-density lipoprotein cholesterol
Vaccination-to-IgG timte: The time interval (in day) between the second vaccination to the date of blood testing for SARS-CoV-2 IgG
