# Supplementary material for: Metabolic syndrome and the immunogenicity of Pfizer–BioNTech vaccine: a cross-sectional study in Japanese healthcare workers
Source: Diabetol Metab Syndr. 2022 Oct 13;14:149. doi: 10.1186/s13098-022-00918-6 (PMC9556286; doi:10.1186/s13098-022-00918-6)
Supplement: Supplementary file 1 — Additional file 1: Table S1. Characteristics of included versus excluded participants. Table S2. Association between MetS and SARS-Cov-2 spike IgG titers. Table S3. Association between MetS and SARS-Cov-2 IgG titers after excluding those with SARS-Cov-2 infection, or comorbid cancer, heart or lung diseases (N = 912). Figure S1. Distribution of SARS-CoV-2 spike IgG titer across vaccination-to-IgG time [file 13098_2022_918_MOESM1_ESM.docx]

| **Table S1. Characteristics of included versus excluded participants** | | |
| --- | --- | --- |
| **Characteristics** | **Excluded participants** | **Included participants** |
| N | 1533 | 946 |
| Age, mean (SD) | 39.1 (12.0) | 36.7 (12.3) |
| Sex (men) | 441 (28.8) | 298 (31.5) |
| Smoking |  |  |
| Non-smoker | 1284 (83.8) | 855 (90.4) |
| Smoker | 249 (16.2) | 91 (9.6) |
| Alcohol consumption |  |  |
| Non-drinker | 594 (38.8) | 355 (37.5) |
| Drinker consuming |  |  |
| < 1 go/day | 676 (44.2) | 442 (46.7) |
| ≥ 1 go/day | 260 (17.0) | 149 (15.8) |
| Missing | 3 |  |
| Leisure time physical activity |  |  |
| Non-engagement | 325 (21.2) | 197 (20.8) |
| < 150 min/week | 1069 (69.8) | 657 (69.5) |
| ≥ 150 min/week | 137 (8.9) | 92 (9.7) |
| Missing | 2 |  |
| Comorbidity (any of the below) | 85 (5.5) | 29 (3.1) |
| Lung disease | 59 (3.8) | 18 (1.9) |
| Heart disease | 19 (1.2) | 5 (0.5) |
| Cancer | 17 (1.1) | 6 (0.6) |
| History of SARS-Cov-2 infection ^a^ | 19 (1.2) | 5 (0.5) |
| Vaccine-to-IgG test days, median (range) ^b^ | 62 (5-103) | 67 (15-103) |
| SARS-Cov-2 spike IgG (AU/mL), median (IQR) | 6394 (6527) | 5588 (6204) |
| Central obesity ^c^ | 176 (22.2) | 187 (19.8) |
| Missing | 739 |  |
| High BP ^d^ | 227 (21.6) | 213 (22.5) |
| Missing | 480 |  |
| High FPG ^e^ | 9 (52.9) | 65 (6.9) |
| Missing | 1516 |  |
| High TG ^f^ | 143 (13.5) | 93 (9.8) |
| Missing | 477 |  |
| Reduced HDL-C ^g^ | 40 (3.8) | 30 (3.2) |
| Missing | 476 |  |
| Values are n (%), unless otherwise stated; SD: standard deviation; IgG: immunoglobulin G;  ^a^ positive result of either polymerase chain reaction test or the measurement of antibodies against SARS-CoV-2 nucleocapsid protein; ^b^ time interval between the second dose of vaccine and the day of blood draw;  ^c^ waist circumference ≥ 90 cm for men, or ≥ 80 cm for women; ^d^ systolic blood pressure (BP) ≥ 130 mmHg, diastolic BP ≥ 85 mmHg or using antihypertensive medication; ^e^ fasting plasma glucose ≥ 100 mg/dL or using antidiabetic medication; ^f^ triglycerides ≥ 150 mg/dL or using lipid-lowering medication; ^g^ high-density lipoprotein cholesterol < 40 mg/dL for men, or < 50 mg/dL for women. | | |

| **Table S2: Association between MetS and SARS-Cov-2 spike IgG titers** | | | | | | | | |
| --- | --- | --- | --- | --- | --- | --- | --- | --- |
| **MetS/ components** | **N** | **SARS-Cov-2 spike IgG** | | | | | | |
|  |  | **Model 1** | | |  | **Model 1** | | |
|  |  | Beta coefficient | P | Adjusted R squared |  | Beta coefficient | P | Adjusted  R squared |
|  | **Metabolic syndrome** | | | | | | | |
| MetS (-) | 895 | 0 |  |  |  | 0 |  |  |
| MetS (+) | 51 | -0.262 | 0.013 | 0.194 |  | -0.230 | 0.025 | 0.317 |
|  | **Number of metabolic syndrome components** | | | | | | | |
| 0 | 572 | 0 |  |  |  | 0 |  |  |
| 1 | 233 | -0.011 | 0.853 |  |  | 0.012 | 0.832 |  |
| 2 | 90 | -0.146 | 0.088 | 0.195 |  | -0.098 | 0.277 | 0.318 |
| 3 | 32 | -0.216 | 0.107 |  |  | -0.131 | 0.32 |  |
| ≥ 4 | 19 | -0.432 | 0.010 |  |  | -0.489 | 0.003 |  |
| MetS: Metabolic syndrome; Model 1: adjusted for age and sex; Model 2: adjusted for age, sex, smoking, alcohol consumption, physical activity, history of SARS-CoV-2 infection, vaccination-to-IgG time, and comorbid cancer, heart and lung diseases. | | | | | | | | |

| **Table S3: Association between MetS and SARS-Cov-2 IgG titers after excluding those with SARS-Cov-2 infection, or comorbid cancer, heart or lung diseases (N = 912)** | | | | | | | | | | |  |
| --- | --- | --- | --- | --- | --- | --- | --- | --- | --- | --- | --- |
| **MetS/ components** | **N** | **SARS-Cov-2 spike IgG** | | | | | | | | |  |
|  |  | **Model 1** | | |  | | **Model 2** | | | |  |
|  |  | GMT (95% CI) | GMR (95% CI) |  | | GMT (95% CI) | | GMR (95% CI) | |  |  |
|  | **Metabolic syndrome** | | | | | | | | | |  |
| MetS (-) | 865 | 5309 (5049, 5582) | 1.00 (ref) |  | | 5051 (4636, 5503) | | | 1.00 (ref) |  |  |
| MetS (+) | 47 | 4020 (3278, 4929) | 0.76 (0.61, 0.93) |  | | 3816 (3136, 4644) | | | 0.76 (0.62, 0.92) |  |  |
|  | **Number of metabolic syndrome components** | | | | | | | | | |  |
| 0 | 552 | 5387 (5046, 5750) | 1.00 (ref) |  | | 5057 (4593, 5568) | | | 1.00 (ref) |  |  |
| 1 | 225 | 5425 (4944, 5952) | 1.01 (0.90, 1.13) |  | | 5217 (4686, 5807) | | | 1.03 (0.93, 1.15) |  |  |
| 2 | 88 | 4678 (4022, 5440) | 0.87 (0.73, 1.03) |  | | 4550 (3882, 5331) | | | 0.90 (0.77, 1.05) |  |  |
| 3 | 30 | 4233 (3282, 5459) | 0.79 (0.60, 1.03) |  | | 4245 (3335, 5404) | | | 0.84 (0.66, 1.07) |  |  |
| ≥ 4 | 17 | 3554 (2548, 4957) | 0.66 (0.47, 0.93) |  | | 3094 (2270, 4218) | | | 0.61 (0.45, 0.84) |  |  |
| P for trend |  |  | 0.006 |  | |  | | | 0.007 |  |  |
| MetS: Metabolic syndrome; GMT: geometric mean titers; GMR: geometric mean ratio; ref: reference; Model 1: adjusted for age and sex; Model 2: adjusted for age, sex, smoking, alcohol consumption, leisure time physical activity, and time interval (in day) between the second vaccination and the day of blood draw | | | | | | | | | | | |

Participants (aged 20-64 years) in the baseline health check-ups (n = 141,478)

Excluded at baseline:

1. Those with missing information on weight or height (n = 1,898)
2. Those with a history of cancer or cardiovascular diseases (n = 2,668)
3. Missing covariates (n = 49,048)
   1. Smoking status [n = 27,511]
   2. Waist circumference [n = 29,051]
   3. Hypertension [n = 3,016]
   4. Diabetes [n = 24,533]
   5. Dyslipidemia [n = 19,445]

Participants included at baseline (n = 87,864)

Those who did not attend any subsequent health examinations, and those did not have any information on mortality or date of return of resignation after long term sick leave from the baseline to March 31, 2019 (n=7,417)

Participants included in the main analyses (n = 80,447 with 457,934 rows of data)


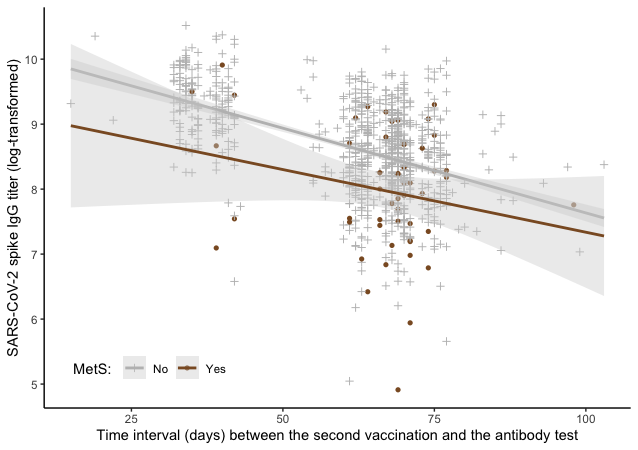


**Figure S1. Distribution of SARS-CoV-2 spike IgG titer across vaccination-to-IgG time**
